# Determinants of Weight Loss in an Interdisciplinary Long-Term Care Program for Childhood Obesity

**DOI:** 10.5402/2012/349384

**Published:** 2012-08-15

**Authors:** A. C. Dubuisson, F. R. Zech, M. M. Dassy, N. B. Jodogne, V. M. Beauloye

**Affiliations:** ^1^Pediatric Endocrinology Unit, Cliniques Universitaires Saint-Luc, Université Catholique de Louvain, 1200 Bruxelles, Belgium; ^2^Department of Internal Medicine, Cliniques Universitaires Saint-Luc, Université Catholique de Louvain, 1200 Bruxelles, Belgium

## Abstract

*Background*. Efforts are needed to improve the long-term efficiency of childhood obesity treatment. To adapt strategies, the identification of subgroups of patients with a greater weight loss may be useful. *Objective.* To analyze the results of a chronic care program for childhood obesity and to determine baseline factors (medical, dietary, and psychosocial) associated with successful weight loss. *Subjects and Method.* We set up a family-targeted and individually adapted interdisciplinary long-term care program. We reviewed the medical files of 144 children (59 boys and 85 girls; 10.5 ± 3.1 y; mean BMI-*z*-score: 2.73 ± 0.62) who had ≥2 interdisciplinary visits and ≥1-year treatment. *Results.* Mean treatment length was 2.2 y (1–6.7 y) with 3 ± 1 visits/year. The duration of treatment did not depend on the initial weight loss, but this was predictive of the weight change over time. Furthermore any additional weight loss was observed with time whatever the initial weight change. High levels of physical activity and daily water intake from baseline conditions were associated with a greater weight loss after 9 months of intervention. In contrast, a high baseline consumption of soft drinks resulted in lower weight loss. Family specific factors such as being a single child or the child's family support were identified as baseline factors which may contribute to better results. *Conclusion.* Our study suggests that the benefit of a chronic weight control program supports the need for its integration into the current concept of treatment. Better prevention policy and parental support may improve the success of the childhood obesity treatment.

## 1. Introduction

Childhood obesity has spread dramatically over the previous decades. To curtail this major health issue, long-term effective weight control programs are essential. In the short term, several studies have shown positive and encouraging outcomes of multidisciplinary approach for childhood obesity. A combination of dietary, physical activity and behavioural interventions compared to standard care or self-help can produce a significant and clinically meaningful reduction in obesity in children and adolescents [1, 2]. This highlights the importance of multidisciplinary programs as the best first-line treatment. However, in most studies, programs are limited to between 6 and 12 months of duration, and beneficial effects are partly lost from 6 to 12 months after completion [3], especially for severely obese children [4]. Long-term follow-up studies of paediatric obesity interventions show a mean 10% reduction in relative weight but also substantial relapse [5, 6]. As obesity is a chronic disease, the question of the need of a chronic care program is raised [7]. Whether the continuation of the program will still be beneficial and how to implement this in a real-life situation remains to be answered.

Moreover, why some children respond differently to obesity treatment remains unclear. Identification of factors associated with better outcomes can help maximize the effectiveness of existing interventions, tailor treatment programs to the specific needs of the patients, and set realistic weight loss goals. Treatment for children presents a unique challenge as nutrition education, physical activity, and behaviour modification must be presented to both the parents/caregiver and child. Parental involvement and individual counselling have been recognised as an important feature of behavioural programs, particularly in preadolescent children [3, 4, 9]. It is thus relevant to examine the impact of family characteristics and psychosocial factors on children's weight loss. 

In this paper, we first analyzed the 5-year results of an interdisciplinary long-term care program for childhood obesity. Secondly, we determined the baseline factors (medical, dietary, and psychosocial) which were associated with weight loss. 

## 2. Subjects and Methods

### 2.1. Intervention Structure

In 2000, we set up an interdisciplinary approach for the treatment of childhood obesity. Our approach is an individually adapted (specific for each patient) family-based, behavioural lifestyle and dietary intervention program. It consists in joint consultations where each child and his family are seen by both a psychologist and a paediatric endocrinologist at the same time. After a time together, the patient is examined (weight, height, blood pressure, and Tanner stage) by the physician in a separate room which gives the opportunity to the child of having personal time with the paediatrician. During this time, the parents/family/caregivers are seen by the psychologist trained in family therapy. Then, the child and the physician get back to the psychologist and the family for a resume of the situation in order to make some decisions. Thereafter, the child and his family are taken by the psychologist to the dietician. The psychologist gives a summary of the situation, and then all the family is seen by the dietician. 

Before each session, the interdisciplinary team reviews the situation of each patient. At the end of the visit a letter, including all the decisions taken with the child and his family, and some personal encouragement for the child is written and sent to the patient one month later. For further analysis, the implementation of those decisions was defined as adherence to the treatment. The patient attends the next appointment, generally every 3 to 6 months. The duration of intervention is determined by the needs of the patient. They participate in our program as long as they want or need. Between two interdisciplinary visits, the patient and his family may meet the dietician or the psychologist individually if needed. Some patients are referred to a specific psychotherapist, and individual physiotherapy can also be prescribed in some situations to reintroduce physical activity especially if joining a sport club is difficult at the beginning of the treatment. The psychotherapy and the physiotherapy are defined in our approach as “adjuvant therapies.” 

### 2.2. Program Orientation

Our way to treat obese children is based on a solution-focused therapy [10]. The team develops realistic goals (small step changes) together with the child and his family rather than imposing ideas and assumptions about what they need to do to change their lifestyle [11]. We start the treatment from their questions or their needs (What can we do for you?) in order to stimulate them to be an active player in their own changes. We focus on the development of the confidence and competence of the parents or caregivers and of the children. 

Our approach is also in agreement with the evidences published in the BMJ [12] by Edmunds et al. in 2001. We encourage the child to “grow without gaining weight” which decreases BMI slowly. The dietician does not prescribe any specific diet but focuses on healthy eating patterns (decrease exposure to obesogenic foods, designate times for family meals, and allocate individual portions) and on increasing the intake of healthy foods. Adolescents who were educated about better food choices of moderate portion sizes had been described to be more successful in the long term than teenagers who were given a structured meal plan or restrictive diets [13]. We propose that the child joins a sports club or a youth organization. But, we mostly encourage regular daily activities such as riding a bicycle, walking the dog, dancing, gardening, using the stairs instead of elevators, and playing outside with friends who are more easily integrated into a child's lifestyle than participation in organized sport teams. Data suggested that less structured, more flexible lifestyle exercise may be superior to more structured and higher-intensity aerobic exercise for weight control [14]. Recommended activities must be enjoyable and consistent with the child's and his/her family's lifestyle and be rewarding irrespective of the health benefits [14]. A complementary approach is also to reduce sedentary free-time activities. A psychologist is present during the interdisciplinary visits. Obesity may be reactive to an event of life (divorce of their parents, difficulties at school…). Parents are encouraged to not focus on weight loss but address their and the child's internal needs by expressing feelings and nurturing the child emotionally. The parents are targeted as the main agents of change, and they are responsible for inducing this change in the family home [9], not specifically at the target child. Extended family members are included as a means of reaching all people who play a significant role in the child's health. 

### 2.3. Subjects and Assessments

428 medical files of children who entered the interdisciplinary consultations between 2002 and 2007 were retrospectively reviewed. Inclusion criteria were [1] to have participated in, at least, 2 interdisciplinary visits and [2] to have at least one year of treatment. Children with obesity due to an organic/syndromic cause or with type 2 diabetes were excluded. Among the included patients: 73% were Caucasian, 12% were Hispanic, 10% were Arabian, 2% were African, 2% were Asiatic (representative of the national population). The latest visit was defined as the most recent visit reported in the medical files when they were reviewed between 2007 and 2009. Thus, for some children the latest visit is the last visit before they were no longer monitored. For other children, the latest visit is not the last because they are still monitored by the team.

Weight was measured (patient in socks with no shoes and wearing a light gown) in kilograms to the nearest 0.1 kg using a medical weight scale (SECA nondigital medical scale), zeroed and calibrated before each weight. A stadiometer (Holtain limited, Crymmych, PEMBS. UK), calibrated in 0.1-cm intervals, was used to determine height. BMI (kg/m^2^) were expressed relative to the Cole population reference data [8]. Weight loss was defined by reduction of the BMI standard deviation score (BMI*-z-*score) since BMI is gender and age dependent in childhood. BMI*-z-*score standardizes an individual's size, adjusting for age and sex, and allows comparison between values on an equivalent basis. Puberty development was scored in the adolescents according to Tanner stages [15].

The medical, dietary, and psychosocial factors characterizing the child and his family at baseline were assessed retrospectively by an external consultant (who was not made aware of the patients weight evolution) by reviewing the records (standard home-made questionnaire) filled in by the team at the first visit. As it is a retrospective and not a prospective study, a semiquantitative (low/intermediate/high) approach was used to evaluate each factor. For food consumption, no/low means not every day or never; intermediate (if applicable) means every day but at a low (1-2) level; yes/high means every day at a high (>2) level. Physical activity means that the child attends a sports club or a youth organization at least twice a week (yes/high), once a week (intermediate) or never (no/low). Delayed puberty was considered when a girl was assessed M1 > 13.5 years or a boy was assessed G1 > 14 years. Obesity in the family means the child has at least one of the two parents who is obese (BMI > 30 kg/m^2^). Bad quality of sleep was assessed by snoring or short sleep duration (<9 h/night). Recent weight gain means a weight gain within the last year. Family encouragement for the project and child's motivation were assessed according to the involvement of the family and of the child in the project (who had the idea to come here? A doctor, the parents, and/or the child himself?). Social integration was assessed as the participation of the child in out-of-school activities. Family encouragement for leisure activities was assessed as activities realised by all the family out of the home.

### 2.4. Statistical Analysis

We use the quasilikelihood estimation [16] with a linear or a logistic canonical link. For the measures of the same patient, we used an autoregressive correlation matrix and computed the covariance matrix by quasi-least-squares (QLSs) [17].

 For the significance of the results, we used the sandwich variance matrix augmented by the correction proposed by Morel et al. [18] which may be evaluated by the normal distribution.

For continuous independent variables, we worked with nonlinear regression by P splines (B splines with penalization [19] of degree 4). Penalisation was chosen to minimize the Akaike information criterion [20]. 

All the significances were expressed as two sided. Significance was taken at *P* value <0.05.

## 3. Results

Out of 428 patients seen between 2002 and 2007, 322 patients (75%) were interested in our interdisciplinary treatment and attended a second visit. Of those, 144 children (45%) (59 boys (41.0%), 85 girls (59.0%); mean age: 10.5 ± 3.1 years (range 4–19 years; 105 (73%) <13 years, 39 (26%) >13 years; mean BMI*-z-*score: 2.73 ± 0.62) had at least a 1 year intervention program and were selected for our study. Mean length of treatment was 2.2 years (1–6.7 years) with an average of 3 ± 1 visits per year. After 24 months, 72 children were still monitored; 14 achieved a 48-month intervention. The length of treatment or assiduity (number of visits) did not depend on the initial weight loss (Δ BMI*-z-*score) between the first, and the second visit (*P* = 0.63). Sex, age and BMI*-z-*score at the first visit did also not influence the length of intervention (*P* = 0.76; *P* = 0.09 and *P* = 0.43 resp.).

Table 1 described the percentage of patients where BMI*-z-*score decreased and was stable or increased at the second visit and at the latest visit. At the second visit (approximately after 3–6 months), BMI*-z-*score was decreased in 53% of the patients, remained unchanged in 31%, and was increased in 16%. At the latest visit, 77% of patients had a stable or decreased weight.

**Figure 1:**
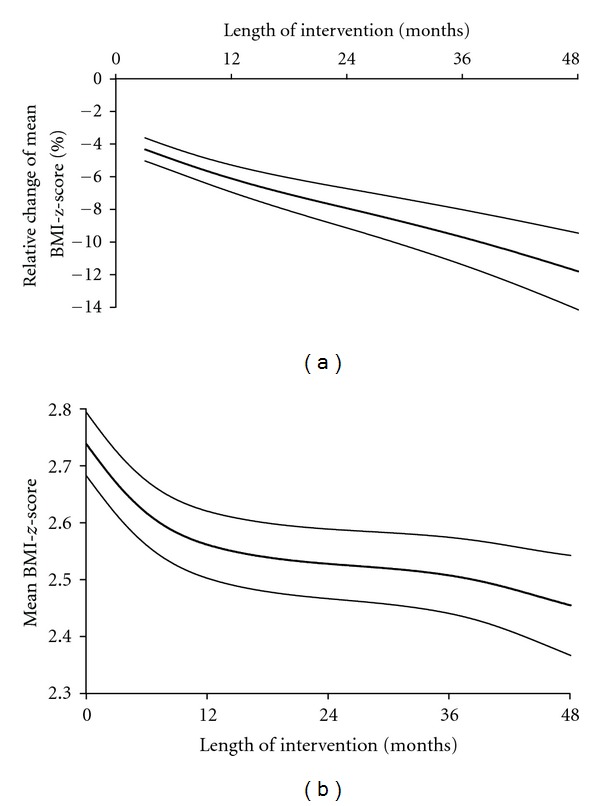
(a) Relative change (%) of mean BMI*-z-*score during intervention and (b) mean BMI*-z-*score during intervention. Data are expressed as mean ± SEM. BMI*-z-*score: body mass index-standard deviation score [8].

**Figure 2:**
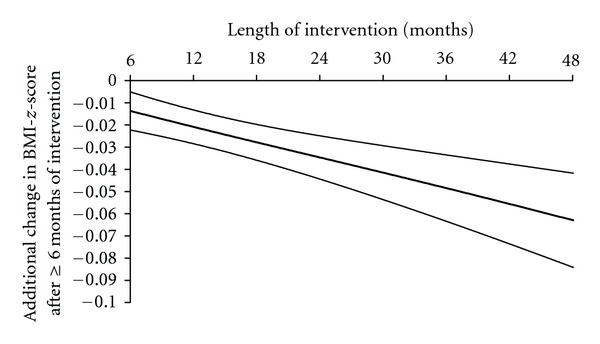
Additional change in BMI*-z-*score observed ≥6-month intervention, controlled for the Δ BMI*-z-*score between the first and the second visit. Data are expressed as mean ± SEM, bivariate analysis.

**Figure 3:**
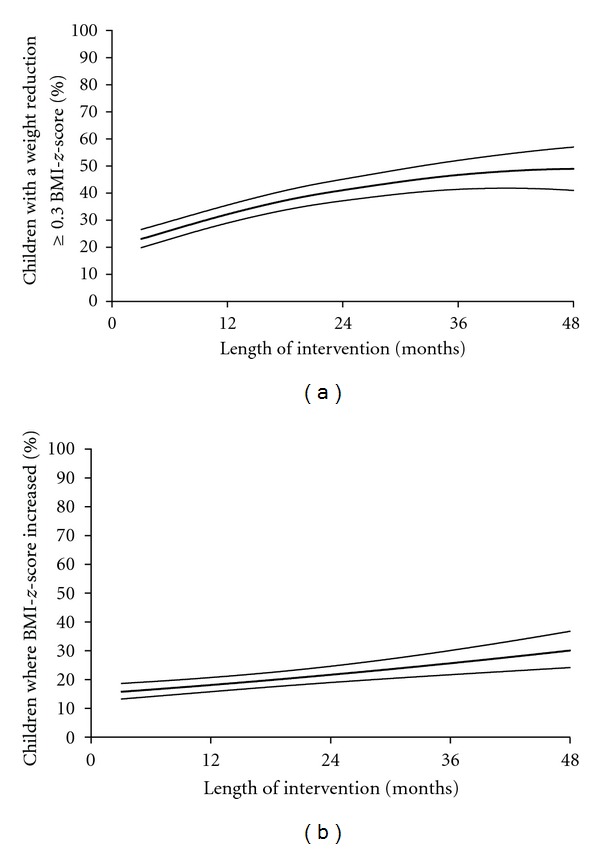
(a) Percentage of children with a BMI*-z-*score reduction ≥0.3 during intervention. Data are expressed as mean (95% CI). (b) Percentage of patients where BMI*-z-*score increased during the intervention (Δ BMI*-z-*score ≥0 at time of intervention in comparison with the initial visit). Data are expressed as mean (95% CI).

**Figure 4:**
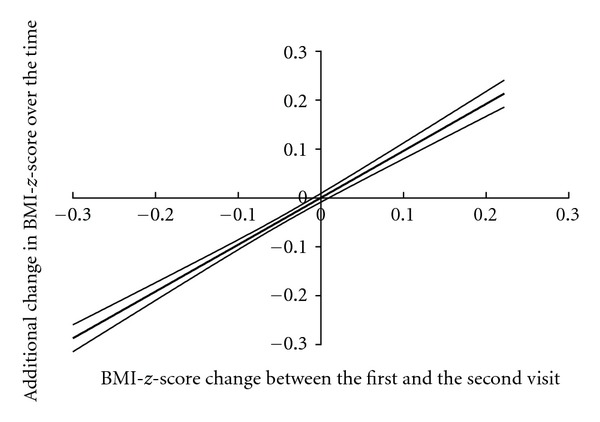
Additional change in BMI*-z-*score obtained in function of the change observed between the first and the second visit, controlled for the length of intervention. Data are expressed as mean ± SEM, bivariate analysis.

In fact, weight loss was mainly observed during the first 6 months of treatment (Figures 1(a) and 1(b)) but was sustained long-term. The mean BMI*-z-*score was decreased by 8% ± 1% (−0.23 ± 0.04 mean BMI*-z-*score) of the initial mean BMI*-z-*score after a mean of intervention of 2.2 years and decreased by 12% ± 2% (−0.28 ± 0.06 mean BMI*-z-*score) for patients with a 48-month treatment. Initial BMI*-z-*score, age at the first visit, sex (data of boys and girls were thus combined for further analysis) and number of visit(s) per year did not influence these results (*P* = 0.73; *P* = 0.27; *P* = 0.95 and *P* = 0.89 resp.). Furthermore, an additional weight loss was observed between 6 and 48 months of intervention whatever the Δ BMI*-z-*score between the first and the second visit (Figure 2). Patients with a BMI*-z-*score reduction ≥0.3 units were 23% (20–27; 95% CI) of the population at 3 months versus 49% (40–58; 95% CI) at 48 months of treatment (Figure 3(a)). However, 16% (13–19; 95% CI) of our patients gained weight after 3 months of treatment. The percentage was increased to 31% (25–38; 95% CI) of the patients reaching 48 months of treatment (Figure 3(b)). The weight change between the first and the second visit was predictive of the additional weight change over the time (Figure 4). 

No evidence of adverse effects on growth, eating disorder pathology or mental health, was found.

We next investigated whether baseline medical, dietary, and psychosocial parameters reported at the first visit could influence the weight change over the time and which one could be associated with weight loss. 

Patients who exercised in daily life before joining the interdisciplinary treatment were the most successful in term of weight loss (Table 2). Preexisting regular physical activity had a statistically significant (*P* = 0.037) positive influence (−0.42 ± 0.11 of mean BMI*-z-*score) on the weight evolution of the child, in comparison with those who did not exercise before starting the treatment (−0.18 ± 0.04 of mean BMI*-z-*score). Having delayed puberty had a negative influence on the evolution of the mean BMI*-z-*score of the patients (−0.02 ± 0.10 of mean BMI*-z-*score) in comparison with those who did not (−0.23 ± 0.03 of mean BMI*-z-*score, *P* = 0.046).

Moreover, baseline daily water intake and daily soda intake had a statistical significant impact on the children's weight outcome (*P* = 0.046 and *P* = 0.00006 resp.) (Table 3). 

We then determined whether the psychosocial context of the child at the first visit may influence the weight change observed later on (Table 4). We showed that being a single child, having family encouragement for the project, the child's motivation, the adherence to the treatment, and the compliance to adjuvant therapies had a statistically positive effect on the mean BMI*-z-*score at 9 months of intervention. The duration of the obesity and dual parent households did not impact the weight changes observed.

## 4. Discussion

This retrospective real-life study reported the outcomes of a long-term approach for treating childhood obesity and identified baseline predictors of weight changes. 

This intervention used interdisciplinary strategies (with effective interaction between the team, not only juxtaposed competences) but had the specificity to be individually adapted with a continuous care program. To our knowledge, this is the first time that the sustained benefit of a chronic intervention is reported and that the feasibility of a long-term intervention in real life is described in obese children. Our results were comparable to results reported by short intervention clinical trials. For example, family-based lifestyle behavioural treatment for obese children with similar clinical characteristics resulted in an average % decrease in overweight of ~7% after 6 months of treatment [3, 4] which is comparable to the 8% decrease observed at 24 months in the current study. However, the duration for the weight change was different. We described only a 4% BMI*-z-*score decrease at the second visit (3-6 months) but a 12% BMI*-z-*score decrease at 48 months. In contrast, in the long term, the results of the abovementioned studies were not as promising as they were immediately after completion of the program (~ −3.5% decrease in weight at 12 months, −1% at 18 months). The beneficial effects of short intervention programs (from 3 to 6 months) were partly lost on the follow-up. Even with a 12-month drug (Orlistat) intervention combined to lifestyle, the initial weight loss was not maintained for more than 6 months [21]. With this emphasis on acute short-term intervention, contemporary healthcare may not be well suited to meet the long-term needs of overweight children and their families fighting against this chronic disease. This indicates the need to develop chronic care models to optimize results, especially for severely obese children [4, 7]. The addition of a 4-month maintenance treatment after short-term weight loss treatment resulted in better maintenance of weight loss compared with the no maintenance group (−0.04 versus +0.05 BMI*-z-*score) but no additional weight loss was obtained over followup [22]. Similar findings were also reported by Reinehr et al. after a 4-year followup [6]. In contrast, our program was still beneficial after 48 months of treatment. Moreover, the percentage of patients with a 0.3 BMI*-z-*score reduction increased over time. At least 50% of children reaching a ≥36-month intervention presented a 0.3 reduction of BMI*-z-*score. 

Longer treatments create challenges in maintaining participants in the program. In adults, the longer the treatment, the greater the proportion of patients who do not attend [23]. This problem may be magnified with families, who may have more challenges in scheduling than individual adults, and where there are multiple people who may want to drop out of the treatment [9]. In our study, 72 participants were still monitored at 24 months. Mean drop-out rates in the literature are varying from 10% to 60% at 12 months of followup [1]. For example, in an Italian multicentric study of nutritional intervention in obese paediatric patients, drop-out rates ranged from 30–34% to 90–94% after 2 years [24]. According to the literature, the main reasons for dropout are loss of interest, relocation, schedule conflict, transport difficulties, family issues for example, limited time for recurrent group sessions, and even for daily household demands [3, 25]. However, even for those patients, encouraging data recently published by Nemet et al. [26] showed that participants in a 3-month brief multidisciplinary intervention still maintained an increased leisure-time physical activity compared to the control group subjects after 1 year of followup. Even the weight benefit was modest after 1 year of followup; this could help them to better general health in the long term. 

The fall in BMI observed in our study may be clinically relevant as demonstrated by many studies, even though not analysed here. Short-term family-based treatment which combined nutrition education, behavioural modification and exercise was shown to improve body composition, lipids profile, blood pressure, and insulin resistance [4, 25, 26]. Many of the obesity-associated complications can be reversed with a 5% decrease in age-adjusted BMI percentile [27]. In adults with impaired glucose tolerance, the Diabetes Prevention Program demonstrated that an intensive lifestyle program that reduced body weight by 7% delayed or prevented the development of type 2 diabetes [28]. Moreover, Savoye et al. [13] reported that obese adolescents with impaired glucose tolerance who were able to limit increases in BMI reverted to normal glucose tolerance 2 years later. Thus, the BMI changes observed over time in our study are likely to be clinically significant as those changes were sustained over the longer term.

We determined parameters characterizing the families and children at baseline conditions which were associated with a better weight control. Indeed, for those less or not responsive patients, new research studies should try to devise new treatments to optimize long-term weight benefits. We demonstrated that those patients could be identified quickly according to the initial weight change observed between the first and the second visits. Tanaka et al. [29] also reported that a greater weight loss between the first and the second visits was a predictive factor in the success of the treatment. Goldschmidt et al. [30] also reported that early weight change seems to be related to treatment response through to the end of the treatment and also the 2-year followup. Identification of factors that promote early weight change is critical because modification of these factors before initiation of the treatment may promote a better early response. 

In our study, similarly to Reinehr et al. [6], reduction of overweight was independent of initial BMI*-z-*score, age at the first visit, and sex. Preexisting regular physical activity contributed significantly to the early treatment response. It is well know that physical activity is related to long-term weight maintenance [31] but, as suggested in another study [32], our data supports its role before the initiation of the treatment. Children with large birth weight, gestational diabetes, no or short-term breastfeeding, parental obesity, asthma, and short sleep duration were described as having an increased risk of obesity [33, 34]. Our analysis suggested that those factors were not determinants for weight loss.

Baseline daily water and soda intake seemed to be a good predictor of early weight change. Consumption of sugar sweetened drinks by adolescents is an independent variable associated with increasing BMI [35], but its role on early weight loss was never examined. Healthy eating habits as eating breakfast and participating in programmed exercises were described to be correlated to healthful BMI, suggesting that these factors may be potentially protective against obesity in 12–16-year old adolescents. Our study extends those results by showing that prevention policy may also be helpful even for children who have to lose weight.

Our results demonstrated that motivated children given family encouragement were more likely to succeed in our treatment. Interestingly, recent reports suggested greater weight loss in obese children when parents alone are targeted for intervention [9], which emphasizes the role of the parents in the child's weight reduction. Moreover, some studies have analyzed the parent's weight changes during the treatment. Larger reductions in adult BMI were associated with more successful results, which indicates that working to enhance the adult role in child weight control programs may improve results [4]. Data from Rhee et al. [36] suggested that parents having an older child, believing that they themselves were overweight, perceiving that their child had a health problem were associated with greater parental readiness to make changes. Emerging research also indicates that overweight children with psychosocial problems or the occurrence of maternal psychopathology are less responsive to weight-control intervention over the longer term [5, 37]. There have been significant lifestyle changes in the family during the previous decades. The divorce rate has increased as well as the number of families with both parents working. Our data suggested that the dual parent households did not affect weight changes observed at 9 months [38]. This is in contrast with a recent study [39] which showed a relationship between single-parents status and excess weight in children. Further studies are needed to explore the dynamics of single-parent households and its influence on childhood diet and obesity. Interestingly, our study showed that a family with an only child may expect a greater weight loss. Other factors [4] such as higher incomes and higher level of education for the mother were also reported to be associated with better results but were not analyzed in the current study.

In conclusion, this study was a first step in determining whether weight loss was achievable with our interdisciplinary approach and highlighted potential success of a continuous care weight control program to lower BMI. An early weight change seems to be a marker for children's long-term treatment response. Preexisting regular physical activity, normal timing of puberty, baseline daily water and soda intake, motivation and some family characteristics predict the early response to the treatment. Better prevention policy and parental support may thus improve the success of the childhood obesity treatment. Our data may provide a better understanding of the factors involved in better weight control and may help to optimize/adapt our strategies for participants who do not benefit from treatment. 

## Figures and Tables

**Table 1:** Evolution of the BMI-*z*-score of the patients during the intervention.

% of patients where BMI-z-score^a^	↓	=	↑
At the 2nd visit (± after 3–6 months)	53%	31%	16%
At the latest visit^b^ (mean = 2.2 y (1–6.7 years))	67%	10%	23%

^a^ BMI-*z*-score: body mass index-standard deviation score [8].

^b^The latest visit is the most recent visit found in medical files when reviewed between 2007 and 2009 (Section 2).

**Table 2:** Influence of medical factors assessed at the first visit on the weight change observed at 9 months of intervention.

Factors studied	No/low	Intermediate	Yes/high	*P* value
Significative				
Physical activity^a^	−0.18 ± 0.04	−0.30 ± 0.05	−0.42 ± 0.11	*P* = 0.037
Delayed puberty^b^	−0.23 ± 0.03		−0.02 ± 0.10	*P* = 0.046
Nonsignificative				
Birthweight (>4000 g)	−0.21 ± 0.03		−0.31 ± 0.11	*P* = 0.37 NS
Gestational diabetes	−0.23 ± 0.04		−0.02 ± 0.14	*P* = 0.15 NS
Breastfeeding (≥6 months)	−0.20 ± 0.04		−0.30 ± 0.07	*P* = 0.21 NS
Obesity in the family^c^	−0.21 ± 0.05		−0.18 ± 0.04	*P* = 0.62 NS
Asthma	−0.21 ± 0.03		−0.37 ± 0.11	*P* = 0.18 NS
Bad quality of sleep^d^	−0.18 ± 0.05		−0.13 ± 0.07	*P* = 0.61 NS

Data are expressed as change in mean BMI-*z*-score ± SEM at 9 months of intervention. *P* < 0.05, significant. NS: not significant.

^a^Physical activity means that the child joins a sport club or a youth organization at least twice a week (yes/high); once a week (intermediate) or never (no/low).

^b^Delayed puberty was considered when a girl was assessed M1 > 13.5 years or a boy was assessed G1 > 14 years.

^c^Obesity in the family means that the child has at least one of the two parents who is obese (BMI > 30 kg/m^2^).

^d^Bad quality of sleep was assessed by snoring or short sleep duration (<9 h/night). This information was not available for all the patients (*n* = 76).

**Table 3:** Influence of dietary factors assessed at the first visit on the weight change observed at 9 months of intervention.

Factors studied	No/low^a^	Intermediate^b^	Yes/high^c^	*P* value
Significative				
Daily water intake	−0.16 ± 0.04		−0.25 ± 0.04	*P* = 0.046
Daily soft drinks intake	−0.38 ± 0.06	−0.15 ± 0.03	+0.08 ± 0.07	*P* = 0.00006
Nonsignificative				
Daily fruits intake	−0.18 ± 0.04	−0.25 ± 0.04	−0.32 ± 0.08	*P* = 0.16 NS
Eating breakfast every day	−0.18 ± 0.05		−0.25 ± 0.04	*P* = 0.22 NS
2 hot meals a day	−0.23 ± 0.04		−0.16 ± 0.06	*P* = 0.29 NS
Daily juice intake	−0.20 ± 0.05	−0.22 ± 0.03	−0.23 ± 0.04	*P* = 0.73 NS
Daily vegetables intake	−0.25 ± 0.04	−0.20 ± 0.04	−0.15 ± 0.07	*P* = 0.31 NS
Daily soup intake	−0.23 ± 0.04	−0.21 ± 0.04	−0.19 ± 0.07	*P* = 0.68 NS
Daily cookies intake	−0.39 ± 0.11	−0.30 ± 0.06	−0.21 ± 0.03	*P* = 0.12 NS
Snacker	−0.15 ± 0.05		−0.24 ± 0.04	*P* = 0.15 NS
Large portions	−0.24 ± 0.05		−0.21 ± 0.04	*P* = 0.55 NS

Data are expressed as change in mean BMI-*z*-score ± SEM at 9 months of intervention. *P* < 0.05, significant. NS: not significant.

^a^Low means not every day or never.

^b^Intermediate means every day but in a low quantity (1-2).

^c^High means every day in a high quantity (>2).

**Table 4:** Influence of psychosocial factors assessed at the first visit on the weight change observed at 9 months of intervention.

Factors studied	No/low	Intermediate	Yes/high	*P* value
Significative				
Only child	−0.19 ± 0.03		−0.36 ± 0.07	*P* = 0.026
Familial encouragement to the project^a^	−0.12 ± 0.04	−0.26 ± 0.04	−0.39 ± 0.08	*P* = 0.0035
Child's motivation^a^	−0.05 ± 0.03	−0.30 ± 0.04	−0.55 ± 0.07	*P* = 0.000000014
Adherence to the treatment^b^	−0.04 ± 0.03	−0.29 ± 0.03	−0.54 ± 0.07	*P* = 0.000000021
Compliance to adjuvant therapies^c^	−0.10 ± 0.04	−0.23 ± 0.03	−0.35 ± 0.06	*P* = 0.0014
Non-significative				
Recent weight gain^d^	−0.17 ± 0.11		−0.22 ± 0.03	*P* = 0.64 NS
Parents at home after school	−0.22 ± 0.05	−0.22 ± 0.04	−0.21 ± 0.09	*P* = 0.91 NS
Dual parents households	−0.24 ± 0.05		−0.20 ± 0.04	*P* = 0.5 NS
Social integration^e^	−0.21 ± 0.04	−0.23 ± 0.04	−0.24 ± 0.08	*P* = 0.74 NS
Familial encouragement to leisure activities^f^	−0.19 ± 0.04	−0.26 ± 0.05	−0.33 ± 0.09	*P* = 0.17 NS

Data are expressed as change in mean BMI-*z*-score ± SEM at 9 months of intervention. *P* < 0.05, significant. NS: not significant.

^a^Familial encouragement to the project and child's motivation were assessed by the team using the involvement of the family and of the child in the project (Section 2).

^b^Adherence to treatment was assessed by the implementation of the decisions taken together (team and family).

^c^Compliance to adjuvant therapies means that the child and his family took part in psychotherapy or in physiotherapy as suggested by the team.

^d^Recent weight gain means a weight gain for less than 1 year.

^e^Social integration was assessed by the team using the participation of the child in extrascholar activities.

^f^Familial encouragement to leisure activities was assessed by the team according to activities realised by all the family out of home.
